# A pathway study of university embeddedness in provincial innovation systems based on field theory: Empirical evidence from China

**DOI:** 10.1371/journal.pone.0344374

**Published:** 2026-03-26

**Authors:** Zhangyuze Wang, Yifei Sun, Lili Gu, Xingguang Ye

**Affiliations:** 1 Party Committee Organization Department, Ningbo University of Technology, Zhejiang Ningbo, China; 2 School of Management, Universiti Sains Malaysia, Penang, Malaysia; 3 School of Logistics and E-commerce, Zhejiang Wanli University, Zhejiang Ningbo, China; 4 School of Language, Universiti Sains Malaysia, Penang, Malaysia; USTC: University of Science and Technology of China, CHINA

## Abstract

This study employs Bourdieu’s Field Theory and fuzzy-set Qualitative Comparative Analysis (fsQCA) to investigate how university-based scientific and technological talent cultivation mechanisms influence Provincial Innovation (PI) capacity in China. Using data from 31 provincial-level regions, two high-performing innovation pathways were identified: (1) a market-driven pathway propelled by technology commercialization and international cooperation; and (2) a government-led pathway defined by robust policy support and institutional coordination. The findings highlight that high-level PI stems from the synergistic integration of HC, ERC, and international scientific exchange. Conversely, four non-high PI pathways reveal that performance is inhibited by resource scarcities, weak institutional frameworks, and ineffective collaboration mechanisms. Those findings indicate that innovation success does not depend on isolated institutional elements, but on the synergy of configurational complementarity Theoretically, this work extends Bourdieu’s Field Theory into the realms of regional innovation and higher education studies. Methodologically, it demonstrates the value of fsQCA in uncovering nonlinear, multi-factor causal mechanisms. Practically, the study offers guidance for localized talent strategies and university reforms aimed at enhancing regional innovation resilience.

## 1. Introduction

In recent years, the global economic system has undergone profound structural transformations. As deglobalization trends intensify and geopolitical tensions escalate, international industrial chains are being restructured at an accelerated pace., This shift has driven global supply chains to pivot from an “efficiency-first” paradigm toward one centered on “security and controllability” [1]. Against this backdrop, the core focus of national innovation systems has gradually moved from “global collaborative advantage” to “local innovation resilience,” positioning provincial-level regions as the primary drivers of national technological self-reliance [2]. China currently sits at the critical intersection of these global trends. On one hand, ongoing policy initiatives continue to advance the “domestic circulation as the mainstay” strategic layout, thereby strengthening provincial autonomy and responsibility within the innovation system [3]. On the other hand, the scale of university-trained scientific and technological talent (STT) —both in terms of local cultivation and the return of researchers from abroad—continues to rise [4,5]. Data from the Ministry of Education shows that in 2023, the number of Chinese returnees reached 580,000, a historical high, reflecting the strong absorption capacity for high-level STT domestically. However, as the international scientific and technological collaboration environment becomes increasingly restrictive and risks of external technological dependence increase, provincial reliance on local STT has intensified. In this evolving landscape, universities have transitioned from being mere intermediary conduits for the central government to becoming locally embedded innovation anchors. Consequently, these institutions now serve as pivotal agents in the structural advancement of provincial science and technology ecosystems [6–8].

Provincial innovation (PI), as a vital bridge connecting national strategic objectives and local implementation capabilities, is becoming a fundamental unit of “technological sovereignty” [9,10]. Provinces possess strong institutional flexibility and policy leverage to respond rapidly to major national scientific and technological deployments [11]. Simultaneously, significant differences exist among provinces in scientific and technological resource allocation, talent policy formulation, and R&D infrastructure development [12]. Their innovation capabilities directly determine the nation’s resilience in coping with global technological decoupling [13,14]. Under central policy directives promoting “innovation-oriented provinces” and “STT-strong provinces,” provincial units are no longer mere “terminal executors” of the innovation system but have become frontlines for technological security and industrial upgrading.

In this context, Cultivating STT in universities has become a key mechanism supporting PI capabilities. Universities are not only providers of high-level human capital but also primary engines of regional knowledge production and catalysts for its transformation [15,16]. By driving industrial upgrading and systemic technological advancement, high-quality STT reinforces the local innovation ecosystem, thereby positioning the university as the essential nexus integrating talent supply, knowledge creation, and regional development requirements. Therefore, STT cultivation plays an indispensable role in strengthening the foundation of provincial innovation and enhancing overall competitiveness [17–19].

In recent years, existing studies have made significant progress in revealing the relationship between STT and PI. On the one hand, research focusing on the spatial patterns of STT has demonstrated that the distribution, concentration, and mobility of high-skilled personnel have a substantial impact on regional innovation performance [20,21]. On the other hand, studies adopting a policy and institutional perspective have emphasized how innovation policies, governance systems, and regional innovation frameworks support talent-driven innovation activities, highlighting the importance of institutional environments [22,23]. Despite the growing body of related research, the current state of provincial-level innovation development in China continues to face numerous challenges. For example, significant regional disparities in innovation performance persist, with eastern coastal provinces maintaining a distinct advantage while the central and western regions continue to trail behind. The innovation output gap across provinces is widening, exacerbated by a heavy concentration of talent and innovation factors in specific hubs. Consequently, this geographic imbalance results in a lack of meaningful interregional innovation synergy

These empirical phenomena indicate that existing research perspectives are still inadequate for capturing the complexity of provincial innovation in China. One line of research tends to conceptualize talent as a measurable stock or flow of human capital, overlooking how universities as key innovation actors cultivate, organize, and transform talent internally. Another line of research often portrays universities as passive policy implementers or homogeneous organizations, neglecting the interactive relationships among university talent cultivation mechanisms, institutional contexts, and knowledge transformation processes [24,25]. Thus, although existing studies have demonstrated the macro-level importance of talent and institutions, they fail to explain how internal university mechanisms transform talent cultivation into regional innovation capacity. In other words, there remains a lack of systematic empirical analysis that reveals how different combinations of factors jointly constitute multiple pathways to high-level innovation, limiting our understanding of regional disparities in innovation performance. Methodologically, prior studies have predominantly relied on linear regression or single-variable analytical approaches that assume independent and linear relationships among factors [25,26]. While such frameworks can identify the effects of individual variables, such as talent, institutions, or resources, on innovation performance, they fail to capture the complex, context-dependent interactions among multiple factors. Therefore, it is necessary to adopt new approaches capable of analyzing multi-factor configurational relationships to more comprehensively uncover how university mechanisms for cultivating STT function within complex innovation systems.

To address the above issues, it is necessary to adopt a theoretical framework that better captures the interactive relationships among multiple actors. In this context, Bourdieu’s Field Theory provides a new analytical lens. Centered on the tripartite structure of “field–capital–habitus,” the theory emphasizes how actors mobilize different forms of capital and form corresponding practice logics within specific institutional structures. This perspective enables universities to be understood as embedded institutional actors in the innovation field, whose talent cultivation activities interact with institutional environments, resource structures, and habitual practices. It therefore more effectively compensates for the limitations of existing studies in explaining how university talent cultivation mechanisms influence regional innovation performance under different institutional configurations.

Accordingly, this paper introduces Bourdieu’s Field Theory to construct a theoretical model analyzing how university STT cultivation impacts PI through the tripartite “field-capital-habitus” structure. Using fuzzy-set Qualitative Comparative Analysis (fsQCA) with China’s 31 provinces as samples, we identify multiple pathways and mechanisms through which university talent cultivation affects PI under different institutional configurations. The contributions of this paper are threefold. Theoretically, it introduces Bourdieu’s Field Theory into the study of university–regional innovation relationships, extending its explanatory power in regional studies and education policy analysis. Methodologically, it demonstrates the strengths of configurational thinking in identifying nonlinearity, equifinality, and conditional complementarity. Empirically, it provides practical insights for formulating region-specific talent policies and advancing university reform.

## 2. Literature review

### 2.1 Bourdieu’s Field Theory

Bourdieu’s Field Theory is a sociological framework for understanding human behavior. It divides society into two objective structures: “primary” and “secondary.” Primary objective structure refers to the means and methods of social resource distribution and utilization, while secondary objective structure refers to the concrete thought patterns and behavioral methods of practical activities [27]. Field Theory specifically comprises three dimensions: field, capital, and habitus. “Field” refers to an “objective network” among various positions [28,29]; “Capital” denotes accumulated foundations within social fields [30]; “Habitus” shares a symbiotic relationship with fields—it originates from fields and manifests as dispositions and behavioral orientations [30,31].

In the field of higher education, Bourdieu’s Field Theory has been widely applied to uncover the power structures and capital competition both within universities and between universities and their broader social environments. Universities are not only spaces for knowledge production and dissemination but also complex fields constituted by the interaction of multiple forms of capital—academic, social, cultural, and economic [32,33]. Scholars employing Field Theory have analyzed academic hierarchies, disciplinary divisions, resource allocation, and evaluation systems within higher education, emphasizing that academic status often stems from unequal capital accumulation [34–36]. Such inequalities are continuously reproduced through habitus. For instance, the academic habitus cultivated by faculty members through research activities influences their scholarly orientations and teaching practices, thereby shaping the institutional academic style and the overall pattern of knowledge production [37].

Moreover, Field Theory has been instrumental in examining the tensions between globalization and localization in higher education [38,39]. As international competition intensifies, universities face increasing pressure to compete for prestige and resources in the global academic field. This process reshapes institutional habitus and capital logic. Some studies suggest that in the pursuit of global rankings and research performance, universities have developed a “logic of academic capital dominance,” which may compromise educational equity, academic autonomy, and the broader mission of talent development [40]. From a field perspective, researchers have also explored how education policies are formulated and implemented differently across institutional contexts: the positions and capital structures of policymakers, institutions, and academic actors determine their interpretations and practices of policy objectives [41,42].

Building on its extensive application in higher education research, Field Theory also provides a valuable analytical framework for understanding the mechanisms of talent cultivation [43]. Within this framework, key actors in the STT cultivation field include universities (core actors), governments, enterprises, and research institutes. Capital elements encompass human resources (faculty) and educational/R&D capital investments. Habitus refers to concrete measures implemented for cultivating STT, including achievement transformation programs, international academic conferences, and collaborative research. While these significantly influence regional STT cultivation, specific impact pathways remain unclear. Therefore, using Field Theory as the theoretical framework allows us to better capture the relational and multi-actor dynamics underlying STT cultivation, particularly the ways in which multiple institutional conditions interact rather than operate independently.

### 2.2 Research perspectives on provincial innovation

Against the backdrop of rapidly evolving global innovation landscapes, regions have become increasingly important functional units within national innovation systems. As a meso-level entity possessing administrative authority, fiscal arrangements, and policy autonomy, the “province” is not only the terminal point of policy implementation but also a frontier for institutional innovation and scientific and technological deployment. PI is widely regarded as an intermediary mechanism connecting national strategies with local resources. Its innovation capability is crucial for enhancing national technological self-reliance and building regional economic resilience [44,45].

Traditional Regional Innovation System (RIS) theory emphasizes synergistic effects among local institutional environments, innovation networks, and knowledge flows [46]. Within this framework, PI systems are seen as outcomes of interactions within the industry-university-government triple helix structure, operating through geographical proximity, trust mechanisms, and institutional embeddedness [47,48]. Recently, Innovation Ecosystem theory further highlights the dynamic evolutionary nature of local innovation, emphasizing the interactive structures and strategic adaptability of multiple actors within complex networks [49,50]. This perspective contends that local innovation capacity depends not only on resource allocation but also on the joint support of institutional evolution, organizational learning, and relational capital [51].

Compared to cities or metropolitan areas, provinces possess not only cross-city resource integration capabilities but also stronger policy tools and governance mobilization capacities, making them crucial spatial units for understanding Chinese-style innovation governance and institutional adaptability. Studying PI helps identify spatial heterogeneity within China’s innovation ecosystems and reveals interactive logics among institutional configurations, technology diffusion, and talent mechanisms.

### 2.3 Talent cultivation and provincial innovation

Talent is a core driver of regional innovation systems, especially amid growing trends of “deglobalization” and “technological localization,” which heighten local dependence on indigenous STT [52]. As primary cultivators of STT, universities are undergoing profound institutional repositioning within PI systems [53]. They are no longer passive knowledge suppliers but increasingly transforming into embedded innovation actors, assuming key roles in coordinating networks, integrating resources, and supporting local innovation systems [54].

A large body of empirical research has provided a solid foundation for understanding the role of universities within regional innovation systems. Overall, existing studies have evolved through three main stages. Early research primarily focused on the spatial agglomeration effect of talent, revealing that the concentration of STT significantly promotes regional knowledge creation and technological innovation, thereby enhancing the absorptive capacity and economic competitiveness of RI [55,56]. As research deepened, scholars began to emphasize the intermediary and hub role of universities [57,58]. Empirical findings indicate that universities serve as core actors in knowledge diffusion and technology transfer within RI networks. Through activities such as research collaboration, university–industry partnerships, technology commercialization, and innovation-oriented education, universities have effectively driven industrial upgrading and innovation synergy. The accumulation of educational and research capital is regarded as a crucial driver of sustained regional innovation growth [59–62]. More recently, studies have further highlighted the structural impact of talent cultivation mechanisms, suggesting that universities, by training, attracting, and reproducing STT, are actively reshaping the knowledge structure and institutional environment of RI. Factors such as educational resource allocation, disciplinary development, research culture, and international collaboration together constitute the core supporting mechanisms of RI [20,21].

Although extensive research has yielded substantial findings in the fields of RI and talent cultivation, scholars remain divided on the mechanisms through which universities exert their influence within innovation systems. Some studies, adopting an institutional-environment perspective, emphasize the decisive role of local government policies, innovation ecosystem construction, and institutional arrangements in shaping the effectiveness of talent, arguing that universities’ innovative performance largely depends on external institutional incentives and resource allocation efficiency [22,23,63]. Others, from an organizational-mechanism perspective, contend that internal factors, such as research culture, academic habitus, resource distribution structures, and governance models constitute the fundamental determinants of universities’ talent innovation capacity and their contribution to regional collaboration [64,65]. In addition, debates over talent agglomeration versus mobility effects have become another focal point: the traditional view highlights how the spatial concentration of STT promotes knowledge spillovers and technological innovation, whereas more recent studies argue that cross-regional mobility, network collaboration, and open knowledge sharing can, under certain conditions, exert even stronger effects on innovation performance [56,66,67]. Overall, while existing research has revealed statistical correlations between talent and innovation, it still lacks an in-depth explanation of how universities, as institutional actors, systematically drive RI capacity through talent cultivation mechanisms within the multidimensional fields of institutions, organizations, and networks.

The above divergence largely stems from limitations in research perspectives and analytical frameworks. On the one hand, many studies tend to simplify RI performance as being driven by a single or a small number of factors—such as institutional environment, internal university mechanisms, or talent agglomeration and mobility—while overlooking the interdependence and mutual embeddedness of these factors [68,69]. This reductionist tendency makes it difficult to explain why the role of universities in RI appears “highly significant in some cases but limited in others.” On the other hand, mainstream empirical approaches are often built upon linear and additive-effect assumptions, treating institutional variables, organizational characteristics, and talent indicators as independent explanatory factors, with little consideration of potential nonlinear effects, threshold effects, or multiple equivalent pathways (equifinality) that may arise from different configurations [25,70]. Under such frameworks, research can identify which factors matter but rarely explain under what contextual and configurational conditions these factors actually interact to produce innovation outcomes [71]. As a result, scholarly understanding of how universities function within RI has remained fragmented and static.

## 3. Methodology

### 3.1 Research framework

PI capability is a critical link for driving new-old kinetic energy conversion and achieving high-quality local economic development. STT, as core resources for knowledge production and technological application, play a driving role in regional innovation systems [53]. Talent agglomeration not only stimulates regional innovation vitality but also helps build localized innovation networks and institutional foundations. Therefore, enhancing university talent cultivation quality and establishing institutionalized talent support mechanisms have become practical pathways for localities to boost innovation capacity.

According to endogenous growth theory, universities serve as vital nodes for knowledge accumulation and technology diffusion, undertaking dual functions of STT education and research innovation. Bourdieu’s Field Theory further provides an analytical framework, emphasizing that actors within specific fields are dually influenced by capital structures and habitus systems, constructing behavioral boundaries through capital operations and cognitive actions [27]. In the regional innovation context, university talent cultivation mechanisms represent crucial processes of capital conversion and habitus perpetuation within the “education field.”

Additionally, the innovation ecosystem perspective highlights multi-actor interactions in local innovation, typically analyzing the “industry-university-government” triple helix structure [47], emphasizing synergistic relationships among universities, governments, and industries. Accordingly, this paper constructs a theoretical model of how STT cultivation influences PI through the “field-capital-habitus” three-dimensional structure.

Specifically in field level: indicators, Industry-University-Research Collaboration (IUCR) and Government Attention to Scientific Talent (GAT), are selected, measuring institutional linkages between universities and external organizations; Capital level: Human Capital (HC) and Educational and Research Capital (ERC) are selected to reflect universities’ resource allocation capabilities in talent cultivation; Habitus level: International Scientific and Technological Exchange (ISE) and Transformation of Scientific and Technological Achievement (TSA) are selected to measure internalization and output capacities of university research activities toward local knowledge systems.

**Fig 1:**
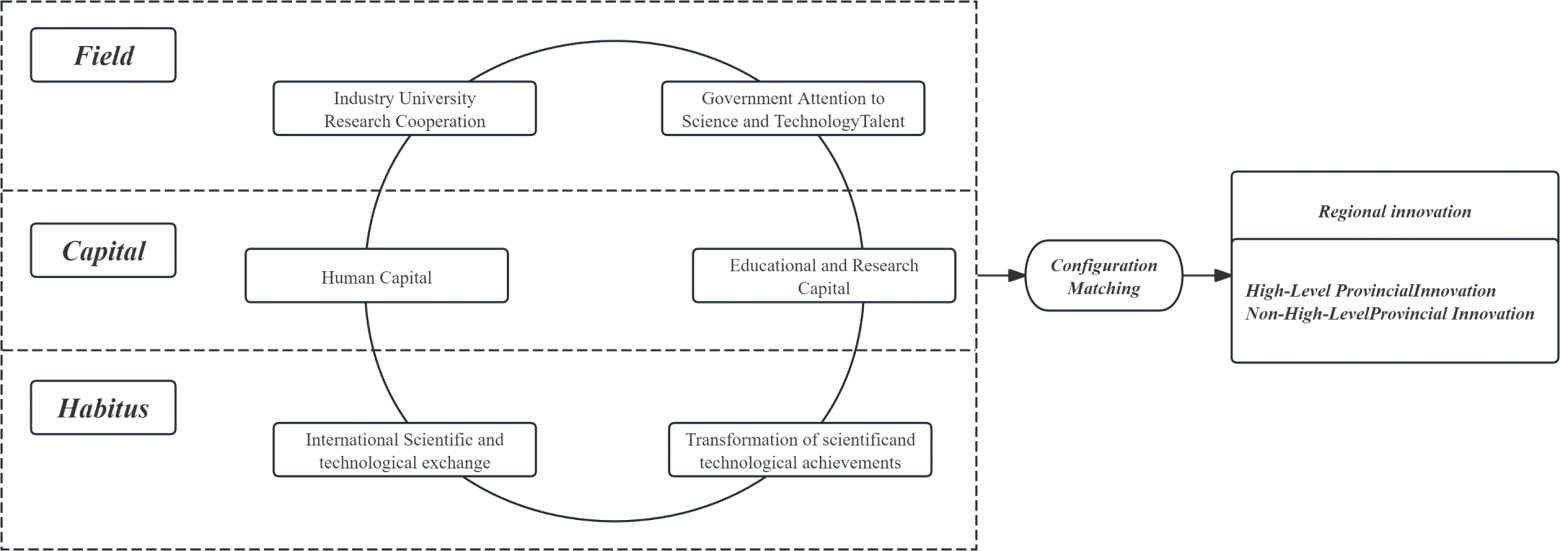
Model of configurational effects.

These six variables constitute the configuration of conditions for the analysis, allowing for an exploration of the equifinal mechanisms through which university talent cultivation influences PI performance, as shown in Fig 1.

### 3.2 Research method

This study adopts the fuzzy-set Qualitative Comparative Analysis (fsQCA) as its primary research tool. Proposed by sociologist [43], the fsQCA method integrates the case-oriented sensitivity of qualitative research with the systematic variable handling of quantitative approaches. This approach effectively identifies the mechanisms through which configurations of multiple conditions drive the outcome, making it particularly suited for meso-level analyses that account for the interdependent effects of various institutional and social factors.

The core idea of QCA lies in treating social phenomena as configurations composed of multiple conditions. By applying set theory and Boolean algebra, QCA identifies the “sufficiency” and “necessity” of various condition combinations in relation to the outcome variable. Currently, QCA has evolved into three types: crisp-set QCA (csQCA), multi-value QCA (mvQCA), and fuzzy-set QCA (fsQCA). Among these, fsQCA allows variables to have membership scores between 0 and 1, thus more accurately reflecting the “partial membership” nature of social variables. This feature gives fsQCA a significant advantage in dealing with gradual variables such as regional institutions, educational investment, and collaborative relationships.

The influence of university-based STT cultivation on PI capacity is characterized by high complexity and diverse pathways. Nonlinear coupling and complementary mechanisms often exist among different conditions. The fsQCA method is well suited to address such a structure, where “multiple conjunctural causation” leads to the same outcome. It can identify various configurational paths leading to high levels of innovation capacity. This study follows the standard fsQCA procedure, including: (1) direct and indirect calibration of variables; (2) necessity analysis (typically based on a consistency threshold of 0.9); (3) generation of the truth table and extraction of complex, parsimonious, and intermediate solutions; and (4) robustness testing and interpretation. This method helps overcome the limitations of traditional linear models and more accurately reveals the multiple pathways through which university talent mechanisms empower PI under different institutional configurations.

### 3.3 Data sources

To ensure both broad representativeness and structural comparability in the fsQCA analysis, the QCA approach emphasizes sample coverage and heterogeneity during case selection (Ragin, 2008). Although the cultivation of STT in universities exerts a generally shared influence on PI capacity, it is also shaped by diverse historical, political, social, and economic factors across regions, resulting in significant regional disparities. Based on this, the present study selects China’s 31 provincial-level administrative units (including autonomous regions and municipalities directly under the central government) as cases for configurational comparative analysis at the provincial level (see supporting documents).

The core data used in this study are derived from authoritative statistical sources, including the China Regional Innovation Capacity Evaluation Report, the Compilation of Science and Technology Statistics of Higher Education Institutions, and provincial Government Work Reports. To ensure the timeliness of data and account for potential time lags in the effect of different indicators, this study adopts the three-year average (2021–2023) of each variable as the final measurement value, following common practices in relevant literature. This approach enhances the stability and temporal validity of the variables.

Specifically, the outcome variable “PI” is sourced from the China Regional Innovation Capacity Evaluation Report. This composite indicator integrates five dimensions: knowledge creation, knowledge acquisition, enterprise innovation, innovation environment, and innovation performance, providing a comprehensive assessment of each province’s innovation capability. Regarding condition variables, IUCR and ERC are also taken from the same report. The former reflects the degree of collaborative innovation networks at the local level, while th e latter measures the capital investment capacity of universities in the talent cultivation process.

The variable GAT, indicating the prioritization of science and technology talent in local policy agendas, is quantified through textual analysis of provincial Government Work Reports from 2021 to 2023. Specifically, the frequency of keywords related to “S&T talent” is used to extract the degree of policy attention. This text-based approach has been widely used to quantify governmental policy attention in official reports [72]. As it captures agenda priority rather than implementation outcomes, any random measurement noise is more likely to weaken the observed associations (i.e., yield conservative estimates) than to generate spurious patterns, therefore is unlikely to alter our main conclusions [73]. Meanwhile, data related to university-based talent—such as HC, ISE, and TSA—are sourced from the Compilation of Science and Technology Statistics of Higher Education Institutions. These indicators cover the number of research personnel, the frequency of international collaboration, and the effectiveness of research commercialization, offering a systematic representation of the resource structure and external connectivity of university talent cultivation mechanisms. It should be acknowledged that the indicators used to measure STT are based on aggregate university-level statistics, which include both locally cultivated and externally recruited personnel.

## 4. Results

### 4.1 Measurement and calibration

Drawing upon Bourdieu’s field theory, the cultivation of STT in universities is not merely a singular educational act but a systemic mechanism shaped by the collaboration of multiple actors—industry, academia, government, and research sectors—alongside capital input and academic cultural production. To identify the causal configurations through which university-based talent cultivation mechanisms influence PI, this study takes PI capacity as the outcome variable and selects six condition variables grounded in Bourdieu’s field theory. Drawing on Seppo & Lilles (2012) and Meng et al. (2024), this study selects IURC and GAT as field-level indicators to capture the structural configuration of the innovation field at the provincial level—respectively reflecting inter-organizational relational patterns and policy orientation [72,74]. Drawing on Okoye et al. (2022), this study selects HC and ERC as capital-level indicators to reflect the availability and mobilization of human and financial resources for talent cultivation [75]. Given Bourdieu’s habitus primarily denotes individual-level internalized dispositions reproduced through practice, it is difficult to measure directly in a province-level study using secondary data and may raise concerns about concept–measurement inconsistency. To address this issue, we draw on educational research that extends the notion of habitus to an institutional/organizational level (institutional habitus), conceptualizing it as stabilized, observable practice patterns embedded in universities’ routines and organizational arrangements [76,77]. Therefore, drawing on Byrd (2018), we operationalize habitus using two indicators that reflect enduring practice orientations: ISE and TSA [78].

Calibration in fsQCA follows the direct method proposed by Ragin (2008), which requires the specification of three qualitative anchors to distinguish full membership, the crossover point, and full non-membership in a set. In the absence of theoretically grounded or substantively agreed-upon absolute thresholds for provincial-level indicators, prior methodological guidelines and applications of configurational comparative methods recommend the use of percentile-based anchors as a pragmatic and theoretically coherent solution [79–81]. Specifically, the 95th percentile of each variable is set as the threshold for full membership (1.0), the 50th percentile as the crossover point (0.5), and the 5th percentile as the threshold for full non-membership (0.0). This approach transforms continuous variables into fuzzy-set membership values.

To avoid ambiguity caused by cases at the 0.5 crossover point, a small constant (0.01) is uniformly added to all cases with a membership score of 0.5. This adjustment enhances the discriminative power of the model. The study covers 31 provincial-level units in China, ensuring both theoretical comprehensiveness and compliance with fsQCA’s requirement for case heterogeneity. The original values, quantile thresholds, and calibration settings of each variable are presented in Table 1.

**Table 1:** Variable description and calibration analysis table.

Condition Variable	Measurement Criteria	Data Source	Variable Calibration		
			Full Non-membership	Crossover Point	Full Membership
PI Capacity	Composite utility value of innovation capacity	《China Regional Innovation Capacity Evaluation Report》	19.585	26.89	52.975
IUCR	Proportion of enterprise funding in internal R&D expenditure of universities and research institutes	《China Regional Innovation Capacity Evaluation Report》	0.105	0.23	0.415
GAT	Degree of government emphasis on technological talent cultivation measured by frequency of related terms in provincial government work reports	《Government Work Reports》	5240.67	6659	9285
HC	Total number of teaching and research staff in each province	《Compilation of Science and Technology Statistics of Higher Education Institutions》	6121.67	35940.33	92187.5
ERC	Educational expenditure (in 100 million RMB) and comprehensive scientific research funding	《China Regional Innovation Capacity Evaluation Report》《Compilation of Science and Technology Statistics of Higher Education Institutions》	568574.6	6548816	26676411
ISE	Total number of participants in collaborative research and international academic conferences	《Compilation of Science and Technology Statistics of Higher Education Institutions》	243.665	2703.33	13922.67
TSA	Patent income and technology transfer income (in thousand RMB)	《Compilation of Science and Technology Statistics of Higher Education Institutions》	3906.165	145704	700394.3

### 4.2Necessity analysis

A configurational analysis was conducted on the conditions of university-based STT cultivation and their influence on PI. As a first step, it is essential to examine whether any of the individual conditions constitute necessary conditions. The consistency threshold for identifying necessary conditions ranges from [0, 1], with a commonly accepted benchmark of 0.900. A condition is considered necessary if its consistency score exceeds this threshold and the corresponding scatter plot shows a concentration on the right-hand side or if more than one-third of the data points lie on the horizontal line at 1.0.

**Fig 2:**
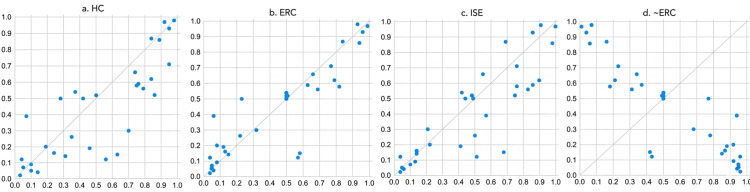
XY plot.

As shown in Table 2 and illustrated by the scatter plots in Fig 2, HC, ERC, and ISE emerge as necessary conditions for achieving high levels of PI. In contrast, under conditions of non-high PI, although the absence of ERC exceeds the 0.900 reference value, its scatter plot does not exhibit the required pattern of right-side concentration or alignment along the horizontal line for more than one-third of cases. Therefore, it does not qualify as a necessary condition.

**Table 2:** Necessity analysis table for individual condition variable.

Condition Variable	High-Level Provincial Innovation		Non-High-Level Provincial Innovation	
	Consistency	Coverage	Consistency	Coverage
IUCR	0.775	0.716	0.562	0.698
~IUCR	0.673	0.533	0.771	0.822
GAT	0.734	0.664	0.592	0.722
~ GAT	0.693	0.558	0.724	0.785
HC	0.924	0.772	0.487	0.548
~HC	0.460	0.400	0.798	0.934
ERC	0.915	0.875	0.414	0.534
~ ERC	0.512	0.394	0.903	0.935
ISE	0.936	0.798	0.493	0.567
~ISE	0.493	0.419	0.825	0.945
TSA	0.889	0.804	0.423	0.516
~ TSA	0.465	0.374	0.839	0.910

In light of these findings, further analysis is required to explore whether there are additional conditions that, while not individually necessary, may jointly contribute to high innovation outcomes. It is also important to examine the synergistic effects of various factors in the context of non-high innovation performance across provinces.

### 4.3Configurational analysis

Configurational analysis reveals the multiple causal pathways formed by combinations of conditions. Following existing studies [82], and given the small-N nature of this study, the frequency threshold was set to 1, the consistency threshold to 0.800, and the Proportional Reduction in Inconsistency (PRI) threshold to 0.700. Based on the output format suggested by [46], as shown in Table 3, the analysis yielded two configurations associated with high-level PI (Table 4), and four configurations associated with non-high-level PI. Each configuration represents a possible causal path.

**Table 3:** Grouping for the realization of high level of PI/non-high level of PI.

Condition Variables	High-Level Provincial Innovation		Non-High-Level Provincial Innovation			
	S1	S2	NS1	NS2	NS3	NS4
IUCR		⊗		⊗	●	●
GAT		●		●	⊗	⊗
HC	●	●	⊗	⊗		●
ERC	●	●	⊗	⊗	⊗	●
ISE	●	●	⊗		⊗	●
TSA	●		⊗	⊗	⊗	⊗
Consistency	0.909	0.982	0.991	0.995	0.99	0.979
Raw Coverage	0.808	0.5	0.712	0.421	0.343	0.237
Unique Coverage	0.343	0.034	0.218	0.019	0.021	0.008
Solution Consistency	0.907		0.987			
Solution Coverage	0.843		0.788			
Representative Provinces	Beijing; Shanghai; Guangdong; Jiangsu; Hubei; Shandong; Zhejiang	Sichuan; Anhui	Qinghai; Xizang; Ningxia; Xinjiang; Gansu; Neimenggu; Guizhou; Yunnan; Shanxi; Guangxi	Xinjiang; Hainan; Gansu	Neimenggu; Hebei	Heilongjiang

● indicates the presence of a core condition;

⊗ indicates the absence of a core condition;

● (hollow circle) indicates the presence of a peripheral condition;

⊗ (hollow circle) indicates the absence of a peripheral condition;

Blank cells indicate that the presence or absence of the condition is irrelevant to the outcome.

**Table 4:** Driving paths and development strategies for high-level PI.

Pathway	Configuration Pattern	Development Strategy
**Technology Achievement Transformation-Oriented**	***Foundational Conditions:*** • Human Capital • Education and Research Capital • International Scientific and Technological Exchange ***Key Mechanism:*** • Transformation of Scientific and Technological Achievements ***Outcome:*** • High-level Provincial Innovation	Leverage technological leadership to build an innovative environment for the transformation of scientific and technological achievements.
**Government-Led Talent Cultivation-Oriented**	***Foundational Conditions:*** • Human Capital • Education and Research Capital • International Scientific and Technological Exchange ***Key Mechanism:*** • Government Attention to Science and Technology Talent ***Outcome:*** • High-level Provincial Innovation	Highlight the government’s leading role to stimulate new vitality in cultivating scientific and technological talent.

The solution consistency for high-level PI is 0.907, indicating that the two configurations account for 90.7% of the cases demonstrating high innovation performance. The solution coverage is 0.843, meaning that these two configurations explain 84.3% of all high-innovation cases. Through the configurational analysis, the study identifies the diverse roles of different conditions in achieving high levels of PI and demonstrates strong explanatory power.

### 4.4Analysis of high-level provincial innovation pathways

**S1: Technology Commercialization-Oriented Talent Cultivation Pathway.**

In this configuration, TSA and ISE serve as the core habitual elements, forming the main innovation-driven pathway alongside ERC. Although there is a lack of effective platforms for IUCR and insufficient policy support, universities leverage abundant resource input and an endogenous research orientation to embed TSA mechanisms throughout the entire talent cultivation process. This enables a closed-loop educational model of “from classroom to laboratory to market.” Crucially, the interaction among TSA, ISE, and ERC forms a mutually reinforcing mechanism: TSA relies on ERC to transform research outputs into practical innovations, while ISE expands the cognitive and cooperative boundaries that enhance the utilization efficiency of ERC and stimulate further transformation activities. Through this feedback cycle, resources, international exchange, and technological achievement transformation continuously strengthen one another, forming a self-propelling system that drives high-level provincial innovation. This pathway highlights the synergistic mechanism between capital input and internationalization, whose essence lies in institutional self-propulsion realized through habitual reinforcement.

Beijing and Hubei represent this pathway. Beijing, in particular, has established a systematic framework for technology commercialization within its universities, effectively institutionalizing the local incubation of research outcomes. It also promotes cross-border mobility of talent and research resources through “Belt and Road” science and technology cooperation. Hubei, despite limited resource coordination, accelerates the flow of research resources into regional industrial systems through policy guidance and laboratory system construction, thereby embedding a results-oriented habitus into its policies. This configuration shows that even in the absence of favorable structural conditions, universities with strong habitual capacity and resource accumulation can still drive a leap in PI capability.

**S2: Government-Led Talent Cultivation Pathway.**

This configuration centers on the GAT, using policy design and resource allocation to compensate for structural deficiencies in IUCR. In contexts where habitual elements are present but collaborative mechanisms are lacking; the government becomes the most dynamic institutional actor in the field. Through specialized talent programs and the provision of financial and institutional resources, GAT interacts synergistically with ERC to enhance universities’ internal capabilities. The government’s intervention not only mobilizes universities’ latent talent supply capacity but also embeds policy incentives into their organizational routines, gradually transforming external policy pressure into internalized institutional habitus. In this way, a policy–resource–habitus interaction mechanism emerges, through which state-led initiatives bridge the structural gap between the educational and regional innovation fields and collectively generate provincial innovation outcomes.

Sichuan and Anhui are typical cases. Facing challenges such as a shortage of high-end talent and mismatched talent structures, Sichuan introduced policies like the “Cuiqing Project” to form a closed-loop system of talent cultivation through institutional selection and targeted funding. Anhui, guided by its “Strengthening the Province through Talent” strategy, restructures university talent training systems around industrial chains and grants universities greater autonomy in talent evaluation and achievement transformation. This configuration illustrates that in regions with immature structural fields, institutional reconstruction driven by the government can become a key external force in upgrading university-based technological talent systems.

Overall, the fsQCA results reveal that multiple configurations can lead to high provincial innovation performance. Although the dominant conditions vary, with some emphasizing technology achievement transformation and others relying on government-led coordination, they converge toward the same outcome. This reflects the principle of *equifinality* in configurational analysis, demonstrating that distinct combinations of field, capital, and habitus can operate as alternative but equally effective routes to innovation success. The findings thus highlight that both endogenous, university-driven mechanisms and exogenous, policy-driven mechanisms can serve as complementary pathways through which universities embed themselves within PI.

### 4.5 Analysis of innovation pathways in non-high-level provinces

**NS1: “Capital–Habitus Deficiency” Pathway**

This configuration reflects a typical dilemma of dual deficiencies in both resources and habitus. In the absence of sufficient ERC, HC, and support for scientific activities, even a certain degree of structural connectivity within the field (e.g., IUCR or GAT) is insufficient to drive high-level PI. This pathway indicates that universities lack the institutional energy to output effectively, talent systems are weak, and the formation of academic habitus is constrained, making it difficult to sustain innovation networks.

Guangxi exemplifies this pathway. Constrained by its economic development level and geographical location, local universities have limited capacity for research investment and scientific output. The system for cultivating STT remains underdeveloped. Although local governments have attempted to introduce resources through reforms such as “joint construction by province and ministry,” the lack of academic habitus and capital foundations has rendered these policies largely ineffective in fostering genuine innovation.

**NS2: Capital Deficiency Pathway**

In this configuration, while there are strong GAT and some willingness to collaborate, the severe lack of ERC and HC input means that the university training system lacks the necessary “energy input” to complete the knowledge production and transformation cycle. Policy focus alone cannot compensate for the material void, and university-industry-research collaboration fails to operate effectively. As a result, the talent mechanisms in universities cannot support PI.

Gansu is a representative case. With a weak economic foundation and limited resource accumulation in universities, the localization of talent and transformation of scientific achievements face multiple obstacles. Although the government has issued measures aimed at optimizing the allocation of scientific resources, the endogenous capability of the research system remains low, and institutional drivers initiated by government intervention have struggled to take root effectively.

**NS3: Habitus Deficiency Pathway**

This pathway presents a scenario of “policy and investment in place, but lacking culture.” Although there is a certain level of ICUR and GAT for talent, universities are weak in TSA and ISE. The immature habitus system prevents research activities from being integrated into local innovation ecosystems. The absence of an educational habitus undermines the application and transformation of knowledge, resulting in a fragmented innovation chain.

Hebei serves as a typical example. Although it has introduced programs to cultivate high-skilled talent and emphasized the central role of universities, a longstanding focus on traditional manufacturing has limited motivation for research transformation. Local universities struggle to establish a habitual pattern of international scientific cooperation, leading to knowledge isolation and talent outflow, which significantly constrains the release of regional innovation capacity.

**NS4: “Field–Habitus Insufficiency” Pathway**

This configuration shows that even if universities have capital support and a foundation for external collaboration, the absence of GAT and the weakness of achievement transformation mechanisms hinder the activation of key institutional dynamics. Without clear goals or incentives, universities as talent cultivation fields struggle to drive breakthroughs in PI.

Heilongjiang is a representative province. Despite a reasonable foundation in research investment and collaboration, the lack of a systemic governmental strategy for STT means that universities cannot effectively embed their research outcomes into the local economy. The transformation mechanisms remain underdeveloped, and university-industry-government collaboration is limited to superficial interactions. As a result, high capital investment fails to translate into high innovation output.

### 4.6 Robustness test

As a set-theoretic approach, fsQCA emphasizes configurational causation, and its results are generally expected to be stable to *reasonable* variations in key analytical choices [83,84]. Following common practice, we assess robustness by adjusting two aspects that may affect solution identification: the PRI consistency threshold and the calibration anchors. Specifically, we first increase the PRI threshold to 0.75 to test whether the identified configurations depend on a more permissive rule for handling contradictory configurations. Under this stricter PRI setting, the resulting configurations remain exactly the same as in the baseline analysis, suggesting that the main solutions are not driven by borderline contradictory cases.

We then examine robustness to alternative calibration stringency by changing the three qualitative anchors used in the direct method. In addition to the baseline anchors (Calibration B: 95th/50th/5th percentiles), we apply a less stringent set of anchors (Calibration A: 75th/50th/25th percentiles). As shown in Table 5, the two dominant configurations for high PI reappear under both calibration schemes, with only modest changes in consistency and coverage values. The solution consistency remains high (above 0.90), and overall coverage remains comparable across specifications. A new configuration emerges under the less stringent calibration; however, its coverage is small, indicating a peripheral pattern that does not materially affect the study’s main conclusions. Overall, the configurational relationships remain largely stable across these parameter perturbations, supporting the robustness of our findings.

**Table 5:** Robustness check under alternative calibration anchors.

Level	Configurational solution (Y1)	Calibration A Coverage	Calibration A Consistency	Calibration B Coverage	Calibration B Consistency
**Configuration**	C1*D1*E1*F1	0.774	0.932	0.808	0.909
	~A1*B1*C1*D1*E1	0.256	0.997	0.5	0.982
	A1B1~C1D1 ~ E1*F1	0.119	0.962	Not observed	
**Solution**	Overall solution	**0.852**	**0.934**	**0.843**	**0.907**

**Note.** Calibration A = 75/50/25 percentiles; Calibration B = 95/50/5 percentiles.

## 5. Conclusions

### 5.1 Research conclusions

This study is based on Bourdieu’s field theory, and from the three-dimensional structure of “field–capital–habitus,” it constructs a theoretical model of how the mechanism for cultivating STT in universities affects PI capacity. Using fsQCA, configuration identification was conducted on relevant data from 31 provinces in China. The study finds that China’s PI capacity generally presents a pattern of both spatial agglomeration and regional disparity. Innovation highlands are mainly distributed in coastal regions such as Beijing-Tianjin-Hebei, the Yangtze River Delta, and the Pearl River Delta, forming an initial “belt-like” innovation distribution pattern. However, there is still a significant imbalance in the allocation of innovation resources, and coordinated governance mechanisms urgently need to be strengthened.

At the level of necessity analysis, HC, ERC, and ISE are identified as fundamental conditions for high-level PI. This indicates that high-quality cultivation of STT depends on stable resource input and sound knowledge connectivity mechanisms. Furthermore, through fsQCA, two typical high-level innovation paths are identified: one is the “achievement-driven” path led by technology commercialization and international cooperation, which emphasizes a results-oriented habitus in the internal research and education mechanisms of universities; the other is the “policy-guided” path with government attention to STT at its core, highlighting the stimulating effect of the policy field on the innovation capacity of universities. These two paths respectively demonstrate how the cultivation of STT can be embedded into the PI system under conditions of habitus advantage or institutional traction.

Meanwhile, the study also identifies four typical non-high-level PI paths, including the “capital–habitus” dual-deficiency type, capital-deficient type, habitus-deficient type, and “field–habitus” insufficient type. These paths all reflect that the absence of a single institutional element cannot be completely substituted by other conditions, indicating that high-level innovation relies on the coordinated configuration of multiple institutional elements. Notably, across all configurations leading to high-level innovation, at least one element of the technical acquisition set—GAT or TSA—emerges as a consistently present condition, alongside the identified necessary factors. This shows that the empowering effect of the talent mechanism in universities is not realized automatically but requires institutional embedding and alignment with cultural habitus to promote the organic linkage of knowledge, resources, and institutions.

In the last, this study not only expands the theoretical understanding of the relationship between talent mechanisms and regional innovation, but also, through configuration analysis, reveals multiple paths at the provincial level, providing strong support for formulating localized talent policies and institutional design.

### 5.2 Research implications

#### 5.2.1 Theoretical implications.

This study makes the following two main contributions at the theoretical level:

First, this paper innovatively introduces Bourdieu’s field theory, breaking through the previous linear logic that viewed the cultivation of STT in universities as a single educational supply process. Starting from the three-dimensional structure of “field–capital–habitus,” it constructs an analytical framework in which universities, as institutional actors, play a role in the PI system. This model not only reveals the multi-level embedded pathways of the university-based STT cultivation mechanism, but also provides a new theoretical perspective for exploring the deep relationship between the education system and regional innovation. Second, in terms of methodology, this study adopts the fsQCA method to identify multiple enabling paths through which university talent mechanisms enhance PI. It demonstrates the applicability of configurational thinking in regional governance and higher education research. Through a comparative analysis of high-level and non-high-level configurations, this paper further verifies the complementarity and asymmetry among institutional variables, offering a new methodological foundation for future research on education and innovation in complex institutional environments. Third, this study helps to bridge the theoretical fragmentation in existing research on regional innovation and university–industry collaboration. Previous studies have often regarded universities as passive knowledge providers or isolated innovation actors, overlooking their relational and structural dynamics within broader institutional contexts [24,85]. By introducing Bourdieu’s field theory, this study extends these discussions by reconceptualizing universities as embedded actors whose practices are shaped by, and in turn reshape, the innovation field. This theoretical refinement provides a more systematic understanding of how universities contribute to regional innovation through talent cultivation, knowledge transformation, and cross-sector collaboration.

#### 5.2.2 Practical implications.

Based on the research conclusions, several recommendations can be proposed.

First, localized and differentiated policy measures should be designed to match the distinct innovation configurations of each province. For regions characterized by limited capital and insufficient institutional support, priority should be given to strengthening university funding systems, improving research infrastructure, and fostering incentive mechanisms that encourage faculty participation in innovation and entrepreneurship. For provinces following a government-led pattern, the focus should shift from policy intensity to policy effectiveness—ensuring that talent policies are implemented consistently and that their long-term impacts are monitored and evaluated. Second, universities should further integrate into local innovation systems by building stronger connections between education, research, and industry. Instead of acting solely as “knowledge providers,” universities should develop joint innovation platforms with enterprises and government agencies, expand international collaboration, and enhance services that support technology transfer and commercialization. Strengthening these links can help universities transform research outputs into tangible regional development outcomes. Finally, coordination among “talent, policy, and industry” should be institutionalized. Local governments are encouraged to improve cross-regional cooperation, share talent resources, and establish joint funding programs for innovation projects. In underdeveloped regions, fiscal support and institutional incentives should be targeted toward improving the spillover effects of university innovation, ensuring that talent cultivation mechanisms contribute more directly to economic and social progress.

### 5.3 Research limitations

This study still has certain limitations. First, the data used in this study cover a three-year period and include both internally cultivated and externally recruited personnel. As such, the indicators reflect the overall talent capacity of universities rather than the specific effects of internal cultivation. However, this relatively short time span may still limit the ability to capture the long-term dynamics of talent cultivation. Future research could incorporate longitudinal or micro-level data to better distinguish between cultivation and recruitment mechanisms and to further explore their differentiated impacts on provincial innovation performance. Second, some key constructs are measured with proxy indicators from secondary data, which may raise construct-validity concerns. For example, text-based measures from official reports capture issue salience in policy discourse rather than policy implementation or outcomes, and may be affected by rhetorical emphasis. Future research could incorporate more direct measures of policy implementation, such as funding allocation records or program-level participation data, to complement text-based indicators of policy attention. Finally, the contextual specificity of this study may limit the generalizability of its findings. The results are most applicable to countries or regions with governance systems similar to China’s—where universities are closely integrated into government-led innovation frameworks. In more market-driven or decentralized contexts, the mechanisms identified here may operate differently. Future research could therefore conduct comparative analyses to test the adaptability of these pathways across diverse institutional settings.

## Supporting information

S1 Data Research data. (XLSX)

## References

[1] Xue J, Li G. Balancing resilience and efficiency in supply chains: Roles of disruptive technologies under Industry 4.0. Front Eng Manag. 2023;10(1):171–6. doi: 10.1007/s42524-022-0247-8

[2] Yan S, Zou L, Growe A, Wang Q. Propositions for place-based policies in making regional innovation systems. Evidence from six high-tech industrial development zones in China. Cities. 2024;154:105322. doi: 10.1016/j.cities.2024.105322

[3] Chen H, Zhang Z, Lin C. How to Enhance Regional Innovation Ecosystem Resilience in China? A Configuration Analysis Based on Panel Data. IEEE Trans Eng Manage. 2024;71:14401–14. doi: 10.1109/tem.2024.3453971

[4] Mukhtarov S, Dinçer H, Baş H, Yüksel S. Policy Recommendations for Handling Brain Drains to Provide Sustainability in Emerging Economies. Sustainability. 2022;14(23):16244. doi: 10.3390/su142316244

[5] Mok KH, Zhang Y, Bao W. Brain drain or brain gain: A growing trend of Chinese international students returning home for development. 2022. p. 245–67. 10.1007/978-981-16-8870-6_11

[6] Natário MMS, Oliveira P. How higher education institutions may catalyse regional innovation ecosystems: The case of polytechnics in Portugal. Industry and Higher Education. 2024;39(3):365–76. doi: 10.1177/09504222241288488

[7] Caniëls MCJ, van den Bosch H. The role of Higher Education Institutions in building regional innovation systems. Papers in Regional Science. 2011;90(2):271–87. doi: 10.1111/j.1435-5957.2010.00344.x

[8] Taxt RE, Robinson DKR, Schoen A, Fløysand A. The embedding of universities in innovation ecosystems: The case of marine research at the University of Bergen. Nor Geogr Tidsskr. 2022;76:42–60. doi: 10.1080/00291951.2022.2041718

[9] Fromhold-Eisebith M. Bridging Scales in Innovation Policies: How to Link Regional, National and International Innovation Systems. European Planning Studies. 2007;15(2):217–33. doi: 10.1080/09654310601078754

[10] Rohe S, Mattes J. What about the regional level? Regional configurations of Technological Innovation Systems. Geoforum. 2022;129:60–73. doi: 10.1016/j.geoforum.2022.01.007

[11] Dansou HD, Carrier M. Decentralization, institutional innovation and governance of inter-territorial relations: A view from Benin. Cities. 2023;133:104115. doi: 10.1016/j.cities.2022.104115

[12] Hu R, Bao Z, Lin Z, Lv K. The Innovative Construction of Provinces, Regional Artificial Intelligence Development, and the Resilience of Regional Innovation Ecosystems: Quasi-Natural Experiments Based on Spatial Difference-in-Differences Models and Double Machine Learning. Sustainability. 2024;16(18):8251. doi: 10.3390/su16188251

[13] Li C, Wang Z. Investigating the Impact of Innovation Policies and Innovation Environment on Regional Innovation Capacity in China. Sustainability. 2024;16(23):10264. doi: 10.3390/su162310264

[14] Zhaoxi H, Lucheng H, Lan M, Hong M. Resilience evaluation of innovation ecosystem in some regions in China from an ecological perspective. Technol Anal Strateg Manag. 2025;:1–18. doi: 10.1080/09537325.2025.2470890

[15] Fonseca L, Nieth L. The role of universities in regional development strategies: A comparison across actors and policy stages. European Urban and Regional Studies. 2021;28(3):298–315. doi: 10.1177/0969776421999743

[16] Benneworth P, Fitjar RD. Contextualizing the role of universities to regional development: introduction to the special issue. Regional Studies, Regional Science. 2019;6:331–8. doi: 10.1080/21681376.2019.1601593

[17] Rossoni AL, de Vasconcellos EPG, de Castilho Rossoni RL. Barriers and facilitators of university-industry collaboration for research, development and innovation: a systematic review. Manag Rev Q. 2023;74(3):1841–77. doi: 10.1007/s11301-023-00349-1

[18] Lee J, Park C. Research and development linkages in a national innovation system: Factors affecting success and failure in Korea. Technovation. 2006;26(9):1045–54. doi: 10.1016/j.technovation.2005.09.004

[19] Klaesson J, Öner Ö, Pennerstorfer D. Getting the first job: Size and quality of ethnic enclaves and refugee labor market entry. J Reg Sci. 2021;61:112–39. doi: 10.1111/jors.12504

[20] Zhang H, Su S, Liu Y. Higher Education Talents Strategy in the Context of Regional Talent Hub Construction: Textual Analysis and Endosymbiotic Cooperation Model. Sage Open. 2023;13(1). doi: 10.1177/21582440231156152

[21] Duan S, Yin F. Interaction Mechanism and Coupling Strategy of Higher Education and Innovation Capability in China Based on Interprovincial Panel Data from 2010 to 2022. Sustainability. 2025;17(15):6797. doi: 10.3390/su17156797

[22] Sun SL, Zhang Y, Cao Y, Dong J, Cantwell J. Enriching innovation ecosystems: The role of government in a university science park. Glob Transit. 2019;1:104–19. doi: 10.1016/j.glt.2019.05.002

[23] Pinto H. Universities and institutionalization of regional innovation policy in peripheral regions: Insights from the smart specialization in Portugal. Regional Science Policy & Practice. 2024;16(1):12659. doi: 10.1111/rsp3.12659

[24] Tolstykh T, Gamidullaeva L, Shmeleva N. Universities as Knowledge Integrators and Cross-Industry Ecosystems: Self-Organizational Perspective. Sage Open. 2021;11(1). doi: 10.1177/2158244020988704

[25] Theeranattapong T, Pickernell D, Simms C. Systematic literature review paper: the regional innovation system-university-science park nexus. J Technol Transf. 2021;46:2017–50. doi: 10.1007/s10961-020-09837-y

[26] Kumar S, Sahoo S, Lim WM, Kraus S, Bamel U. Fuzzy-set qualitative comparative analysis (fsQCA) in business and management research: A contemporary overview. Technological Forecasting and Social Change. 2022;178:121599. doi: 10.1016/j.techfore.2022.121599

[27] Bourdieu P. Distinction a social critique of the judgement of taste. Inequality. Routledge. 2018.

[28] Ignatow G, Robinson L. Pierre Bourdieu: theorizing the digital. Inf Commun Soc. 2017;20:950–66. doi: 10.1080/1369118X.2017.1301519

[29] Wacquant L. Following Pierre Bourdieu into the field. Ethnography. 2004;5:387–414. doi: 10.1177/1466138104052259

[30] Power EM. An Introduction to Pierre Bourdieu’s Key Theoretical Concepts. Journal for the Study of Food and Society. 1999;3(1):48–52. doi: 10.2752/152897999786690753

[31] Asimaki A. Habitus: An attempt at a thorough analysis of a controversial concept in Pierre Bourdieu’s theory of practice. Soc Sci. 2014;3:121. doi: 10.11648/j.ss.20140304.13

[32] Gentry AN, Martin JP, Douglas KA. Social capital assessments in higher education: a systematic literature review. Front Educ (Lausanne). 2025;9. doi: 10.3389/feduc.2024.1498422

[33] Bocquet R, Cotterlaz‐Rannard G, Ferrary M. How Do Prestigious Universities Remain at the Summit: A Bourdieusian View of their Business Models. British J of Management. 2024;35(4):2122–36. doi: 10.1111/1467-8551.12819

[34] Lessky F. Educational inequality and equitable transformation: Combining Bourdieu’s relational theory and the ‘conduct of everyday life’ concept to illuminate underrepresented students’ experiences and success in higher education. Österreich Z Soziol. 2025;50(1). doi: 10.1007/s11614-025-00611-8

[35] Kosunen S. The homology between the private and the public fields in higher education. Br J Sociol Educ. 2023;44:276–90. doi: 10.1080/01425692.2022.2145930

[36] Zhang B. Habitus in motion: a critical appraisal of Bourdieu’s relevance to cross-border higher education. High Educ. 2025. doi: 10.1007/s10734-025-01499-9

[37] McLaughlin S. Using Bourdieu’s concept of habitus to explore higher education decision-making for working class women on an access to higher education course. Studies in the Education of Adults. 2024;58(1):138–56. doi: 10.1080/02660830.2024.2374617

[38] Hwami M, Bedeker M. Social stratifying Kazakhstan: A Bourdieusian social reproduction analysis of higher education internationalisation. Br J Sociol Educ. 2024;45:210–29. doi: 10.1080/01425692.2023.2299966

[39] Khan Eusafzai HA. Educational capital and international mobility: A bourdieusian inquiry into choosing peripheral higher education destination. Social Sciences & Humanities Open. 2024;10:100916. doi: 10.1016/j.ssaho.2024.100916

[40] Lessky F, Nairz‐Wirth E, Feldmann K. Informational capital and the transition to university: First‐in‐family students’ experiences in Austrian higher education. Eur J Educ. 2021;56:27–40. doi: 10.1111/ejed.12437

[41] Pan P, Mu GM. ‘University matching’ in state-regulated Sino-Australian transnational higher education: a Bourdieusian social network analysis. Compare: A Journal of Comparative and International Education. 2024;55(6):1051–68. doi: 10.1080/03057925.2024.2352832

[42] Wong Y-L, Liao Q. Cultural capital and habitus in the field of higher education: academic and social adaptation of rural students in four elite universities in Shanghai, China. Cambridge Journal of Education. 2022;52(6):775–93. doi: 10.1080/0305764x.2022.2056142

[43] Ferrare JJ, Apple MW. Field theory and educational practice: Bourdieu and the pedagogic qualities of local field positions in educational contexts. Cambridge Journal of Education. 2015;45(1):43–59. doi: 10.1080/0305764x.2014.988682

[44] Rodríguez‐Pose A, Wilkie C. Innovating in less developed regions: What drives patenting in the lagging regions of Europe and North America. Growth and Change. 2018;50(1):4–37. doi: 10.1111/grow.12280

[45] Asheim BT. Smart specialisation, innovation policy and regional innovation systems: what about new path development in less innovative regions?. Innovation: The European Journal of Social Science Research. 2018;32(1):8–25. doi: 10.1080/13511610.2018.1491001

[46] Isaksen A, Jakobsen S-E, Njøs R, Normann R. Regional industrial restructuring resulting from individual and system agency. Innovation: The European Journal of Social Science Research. 2018;32(1):48–65. doi: 10.1080/13511610.2018.1496322

[47] Vaivode I. Triple Helix Model of University–Industry–Government Cooperation in the Context of Uncertainties. Procedia - Social and Behavioral Sciences. 2015;213:1063–7. doi: 10.1016/j.sbspro.2015.11.526

[48] Rodrigues C, Melo AI. The Triple Helix Model as Inspiration for Local Development Policies: An Experience‐Based Perspective. Int J Urban Regional Res. 2012;37(5):1675–87. doi: 10.1111/j.1468-2427.2012.01117.x

[49] Granstrand O, Holgersson M. Innovation ecosystems: A conceptual review and a new definition. Technovation. 2020;90–91:102098. doi: 10.1016/j.technovation.2019.102098

[50] Boyer J. Toward an Evolutionary and Sustainability Perspective of the Innovation Ecosystem: Revisiting the Panarchy Model. Sustainability. 2020;12(8):3232. doi: 10.3390/su12083232

[51] Grillitsch M, Sotarauta M. Trinity of change agency, regional development paths and opportunity spaces. Progress in Human Geography. 2020;44:704–23. doi: 10.1177/0309132519853870

[52] Chen S, Sun J, Liang Y. The impact on knowledge transfer to scientific and technological innovation efficiency of talents: analysis based on institutional environment in China. Technol Anal Strateg Manag. 2024;36:1398–413. doi: 10.1080/09537325.2022.2093710

[53] Thomas E, Faccin K, Asheim BT. Universities as orchestrators of the development of regional innovation ecosystems in emerging economies. Growth and Change. 2020;52(2):770–89. doi: 10.1111/grow.12442

[54] Tang Z. Research on cultivation of innovative talents in colleges and universities based on fuzzy evaluation model. Wirel Commun Mob Comput. 2022;2022:1–9. doi: 10.1155/2022/6373351

[55] Jian X, Du D, Liang D. Scale or effectiveness? The nonlinear impact of talent agglomeration on high-quality economic development in China. Heliyon. 2024;10(9):e30121. doi: 10.1016/j.heliyon.2024.e30121 38707458 PMC11066640

[56] Yan L, Fan S, Mengyu L. Innovative talent agglomeration, spatial spillover effects and regional innovation performance-Analyzing the threshold effect of government support. PLoS One. 2024;19(10):e0311672. doi: 10.1371/journal.pone.0311672 39480868 PMC11527277

[57] Rinkinen S, Konsti-Laakso S, Lahikainen K. University as an opportunity space enabler in a regional entrepreneurial ecosystem. European Planning Studies. 2023;32(5):1010–28. doi: 10.1080/09654313.2023.2246522

[58] Thomas E, Faccin K, Asheim BT. Universities as orchestrators of the development of regional innovation ecosystems in emerging economies. Growth and Change. 2020;52(2):770–89. doi: 10.1111/grow.12442

[59] Gustina A, Nurmasari ND, Liu JSC. Open innovation between university-industry: A review of research trends and practices. Journal of Open Innovation: Technology, Market, and Complexity. 2024;10(4):100419. doi: 10.1016/j.joitmc.2024.100419

[60] Rossoni AL, de Vasconcellos EPG, de Castilho Rossoni RL. Barriers and facilitators of university-industry collaboration for research, development and innovation: a systematic review. Manag Rev Q. 2023;74(3):1841–77. doi: 10.1007/s11301-023-00349-1

[61] Hailu AT. The role of university–industry linkages in promoting technology transfer: implementation of triple helix model relations. J Innov Entrep. 2024;13:25. doi: 10.1186/s13731-024-00370-y

[62] Jiang J, Zhao Y, Feng J. University–Industry Technology Transfer: Empirical Findings from Chinese Industrial Firms. Sustainability. 2022;14:9582. doi: 10.3390/su14159582

[63] Zenkienė L, Leišytė L. Strengthening university capacity in regional innovation ecosystem through the participation in the European Universities initiative. European Journal of Higher Education. 2024;14(sup1):88–108. doi: 10.1080/21568235.2024.2410358

[64] Fussy DS. Cultivating a research culture in Tanzanian higher education. Education Inquiry. 2024;17(1):130–51. doi: 10.1080/20004508.2024.2342012

[65] Kienast S-R. How do universities’ organizational characteristics, management strategies, and culture influence academic research collaboration? A literature review and research agenda. Tert Educ Manag. 2023;29(2):139–60. doi: 10.1007/s11233-022-09101-y

[66] Dongling W, Yuming Z, Xinmin L, Chen J, XiaoYi Z, Chang H. Can inter-organizational knowledge-sharing improve enterprise innovation performance? The mediator effect of innovation capability and the moderator effect of network characteristics. Front Commun (Lausanne). 2022;7. doi: 10.3389/fcomm.2022.856301

[67] Wu J, Wu X, Zheng H, Wang T. The scene logic of innovative talent agglomeration: An empirical study based on 54 cities in China. Sustainability. 2024;16:7951. doi: 10.3390/su16187951

[68] Zhao S, Wang J. Proximity and regional innovation performance: the mediating role of absorptive capacity. JSTPM. 2024;16(6):1094–110. doi: 10.1108/jstpm-12-2022-0208

[69] Lin B, Zhang A. Digital finance, regional innovation environment and renewable energy technology innovation: Threshold effects. Renewable Energy. 2024;223:120036. doi: 10.1016/j.renene.2024.120036

[70] Han W, Wang P, Jiang Y, Han H. Nonlinear influence of financial technology on regional innovation capability: Based on the threshold effect analysis of human capital. Sustainability. 2022;14:1007. doi: 10.3390/su14021007

[71] Ragin CC. Fuzzy-Set Social Science. https://books.google.com.hk/books?hl=zh-CN&lr=&id=nZC2dLUH-OAC&oi=fnd&pg=PA1&dq=Charles+Ragin&ots=9QnO_ZBz4y&sig=WQTcQl13uIgRcA-8pGKWD-robws&redir_esc=y#v=onepage&q=Charles%20Ragin&f=false. University of Chicago Press; 2000.

[72] Meng X, Kong F, Fu H, Li S, Zhang K. Is more always better? How government ecological attention influences corporate environmental responsibility: Empirical evidence from Chinese listed companies. Ecol Indic. 2024;159:111686. doi: 10.1016/j.ecolind.2024.111686

[73] Wooldridge JM. Econometric analysis of cross section and panel data. MIT Press. 2010.

[74] Seppo M, Lilles A. Indicators measuring university-industry cooperation. Discussions on Estonian Economic Policy. 2012;20:204.

[75] Okoye K, Nganji JT, Escamilla J, Fung JM, Hosseini S. Impact of global government investment on education and research development: A comparative analysis and demystifying the science, technology, innovation, and education conundrum. Glob Transit. 2022;4:11–27. doi: 10.1016/j.glt.2022.10.001

[76] Schirone M. Field, capital, and habitus: The impact of Pierre Bourdieu on bibliometrics. Quantitative Science Studies. 2023;4:186–208. doi: 10.1162/qss_a_00232

[77] Reay D, David M, Ball S. Making a Difference?: Institutional Habituses and Higher Education Choice. Sociological Research Online. 2001;5(4):14–25. doi: 10.5153/sro.548

[78] byrd derria. Uncovering Hegemony in Higher Education: A Critical Appraisal of the Use of “Institutional Habitus” in Empirical Scholarship. Review of Educational Research. 2018;89(2):171–210. doi: 10.3102/0034654318812915

[79] Wagemann C. Qualitative Comparative Analysis (QCA) and Set Theory. Oxford Research Encyclopedia of Politics. Oxford University Press. 2017. doi: 10.1093/acrefore/9780190228637.013.247

[80] Rihoux B, Ragin CC. Configurational comparative methods: Qualitative comparative analysis (QCA) and related techniques. Sage. 2009.

[81] Ragin CC, Strand SI, Rubinson C. User’s guide to fuzzy-set/qualitative comparative analysis. University of Arizona. 2008.

[82] Fiss PC. Building Better Causal Theories: A Fuzzy Set Approach to Typologies in Organization Research. AMJ. 2011;54(2):393–420. doi: 10.5465/amj.2011.60263120

[83] Zschoch MA. Configurational comparative methods: Qualitative comparative analysis (QCA) and related techniques. Canadian Journal of Political Science. 2011;44(4):743–6. doi: 10.1017/S0008423911000709

[84] Du Y, Kim PH. One size does not fit all: Strategy configurations, complex environments, and new venture performance in emerging economies. J Bus Res. 2021;124:272–85. doi: 10.1016/j.jbusres.2020.11.059

[85] Santini MAF, Faccin K, Balestrin A, Volkmer Martins B. How the relational structure of universities influences research and development results. J Bus Res. 2021;125:155–63. doi: 10.1016/j.jbusres.2020.12.018

